# Two new species of *Athenaea* Sendtn. (Solanaceae) from the Atlantic forests of south-eastern Brazil

**DOI:** 10.3897/phytokeys.178.64609

**Published:** 2021-05-18

**Authors:** Izabella Martins da Costa Rodrigues, Sandra Knapp, João Renato Stehmann

**Affiliations:** 1 Instituto Federal do Espírito Santo, Campus Barra de São Francisco, Av. Herculano Fernandes de Jesus, 111, 29800-000, Irmãos Fernandes, Barra de São Francisco, ES, Brazil Instituto Federal do Espírito Santo Barra de São Francisco Brazil; 2 Department of Life Sciences, Natural History Museum, Cromwell Road, London SW7 5BD, UK Natural History Museum London United Kingdom; 3 Instituto de Ciências Biológicas, Departamento de Botânica, Universidade Federal de Minas Gerais – UFMG, Avenida Antônio Carlos 6627, 31270-901, Belo Horizonte, MG, Brazil Universidade Federal de Minas Gerais Belo Horizonte Brazil

**Keywords:** Alto da Serra, *
Aureliana
*, Brazilian Atlantic Forest, Mata Atlântica, Physalideae, taxonomy, Withaninae, Alto da Serra, *
Aureliana
*, floresta atlântica, Mata Atlântica, Physalideae, taxonomia, Withaninae

## Abstract

Two new species of *Athenaea* Sendtn. (Solanaceae) from the Brazilian Atlantic Forest are described and illustrated. *Athenaea altoserranae* I.M.C. Rodrigues & Stehmann, **sp. nov.** from the Serra do Mar range, in São Paulo State and *Athenaea hunzikeriana* I.M.C. Rodrigues & Stehmann, **sp. nov.** from a restricted area in the north-eastern region of Minas Gerais State and the southern part of Bahia State. Both species have brown to purple-brown or purple mature fruits, a character not found in other species of *Athenaea*. Descriptions, illustrations, complete specimen citations and maps of both species are provided. A dichotomous key to all species of *Athenaea* is also presented.

## Abstract (es)

Duas espécies novas de *Athenaea* Sendtn. (Solanaceae) da floresta atlântica brasileira são descritas e ilustradas. *Athenaea altoserranae* I.M.C. Rodrigues & Stehmann, **sp.nov.** conhecida apenas da floresta atlântica da Serra do Mar, no estado de São Paulo, e *Athenaea hunzikeriana* I.M.C. Rodrigues & Stehmann, **sp.nov.**, conhecida somente de uma área restrita na região nordeste do estado de Minas Gerais e sul da Bahia, divisa entre os estados. Ambas as espécies apresentam frutos amarronzados a arroxeados, um caractere até então não observado em outras espécies de *Athenaea*. São disponibilizadas descrições, ilustrações, citações completas de espécimes e um mapa de distribuição. Uma chave dicotômica de identificação para todas as espécies de *Athenaea* também é apresentada.

## Introduction

*Athenaea* Sendtn. (Solanaceae) is a small, exclusively Neotropical genus with highest species diversity in south-eastern Brazil (Rodrigues et al. 2019). It comprises 12 species of shrubs or small trees mostly growing in forest understorey or openings in highly fragmented Atlantic forests (Rodrigues et al. 2019). Recent re-circumscription of the genus (Rodrigues 2013; Zamberlan et al. 2015) meant that all species of the previously recognised *Aureliana* Sendtn. were transferred to *Athenaea* (Rodrigues et al. 2019).

The genus is characterised by axillary inflorescences, stellate, actinomorphic flowers with five stamens and fruits that are fleshy berries, borne on erect or spreading pedicels. The fruiting calyx is variously accrescent (Rodrigues 2013), ranging from not enlarging at all in fruit to completely enclosing the berry (see Zamberlan et al. 2015 for illustrations of the states). The genus was previously considered a member of the subtribe Withaninae of the tribe Physalideae, based on molecular phylogenetic studies (Olmstead et al. 2008). Recent work, however, has shown that Withaninae is not monophyletic (Deanna et al. 2019); *Athenaea* and two European species of *Withania* Pauq. (*W. frutescens* (L.) Pauq. and *W. aristata* Pauq. from the Mediterranean region and Canary Islands, respectively) are sister to the Andean genera *Deprea* Raf. and *Cuatresia* Hunz., while the rest of *Withania* species resolve as more closely related to *Physalis* L. and relatives (see Deanna et al. 2019 for clade composition).

As part of on-going work producing a monographic treatment of *Athenaea* in this new circumscription, new taxa have been discovered and are described here. *Athenaea altoserranae* I.M.C. Rodrigues & Stehmann (described here) was included in the molecular phylogenetic studies of Zamberlan et al. (2015) and resolved as distinct (as *Aureliana* sp. nov.), but was not given a name at that time (Zamberlan et al. 2015; GenBank numbers in Suppl. material 1). These new taxa can be included in future studies of both morphology and biogeography of the family Solanaceae.

## Materials and methods

Species circumscriptions are based on a combination of morphological (Rodrigues 2013) and molecular (Zamberlan et al. 2015) information. Descriptions are based on observations and data taken from specimens collected in the field between 2003 and 2014. Examination of herbarium specimens from 15 herbaria (acronyms according to Thiers 2019+) http://sweetgum.nybg.org/science/ih/): BHCB, BM, CEPEC, CEN, CORD, ESA, IAC, MBM, MBML, MO, NY, R, RB, SP and SPF; and specimens accessed as digital images via INCT Herbário Virtual (http://inct.splink.org.br) and Reflora – Herbário Virtual (http://reflora.jbrj.gov.br/reflora/herbarioVirtual/) databases were conducted to confirm the novelty of the species described here. All barcode and accession numbers, as well as label details for the new species, are presented in Suppl. material 2 and on the Natural History Museum Data Portal (https://doi.org/10.5519/zc80n093). In the field, fresh flowers were fixed in alcohol to permit detailed descriptions and illustrations using dissection and compound microscopy.

We calculated the Extent of Occurrence (EOO) and Area of Occupancy (AOO) using GeoCat (http://geocat.kew.org) calibrated with the standard 2 km^2^ cell width for the AOO measurement. A preliminary conservation status was assigned using the IUCN (2019) criteria implemented in GeoCat analyses (Bachman et al. 2011) combined with our field knowledge of habitats and threats to the Brazilian Atlantic forests.

## Taxonomic treatment

### Athenaea altoserranae

Taxon classificationPlantaeSolanalesSolanaceae

I.M.C. Rodrigues & Stehmann sp. nov.

2D2E9ABD-FFB1-519F-B394-0971FF1F694B

urn:lsid:ipni.org:names:77217158-1

Figs 1 , 2 , 3

#### Diagnosis.

Similar to *Athenaea fasciculata* from which it can be distinguished in leaves arranged in clusters at the top of the stems (versus evenly distributed), warty somewhat lenticellate floral pedicels (versus without lenticels) and dark purplish-brown mature fruits (versus green).

**Figure 1.**
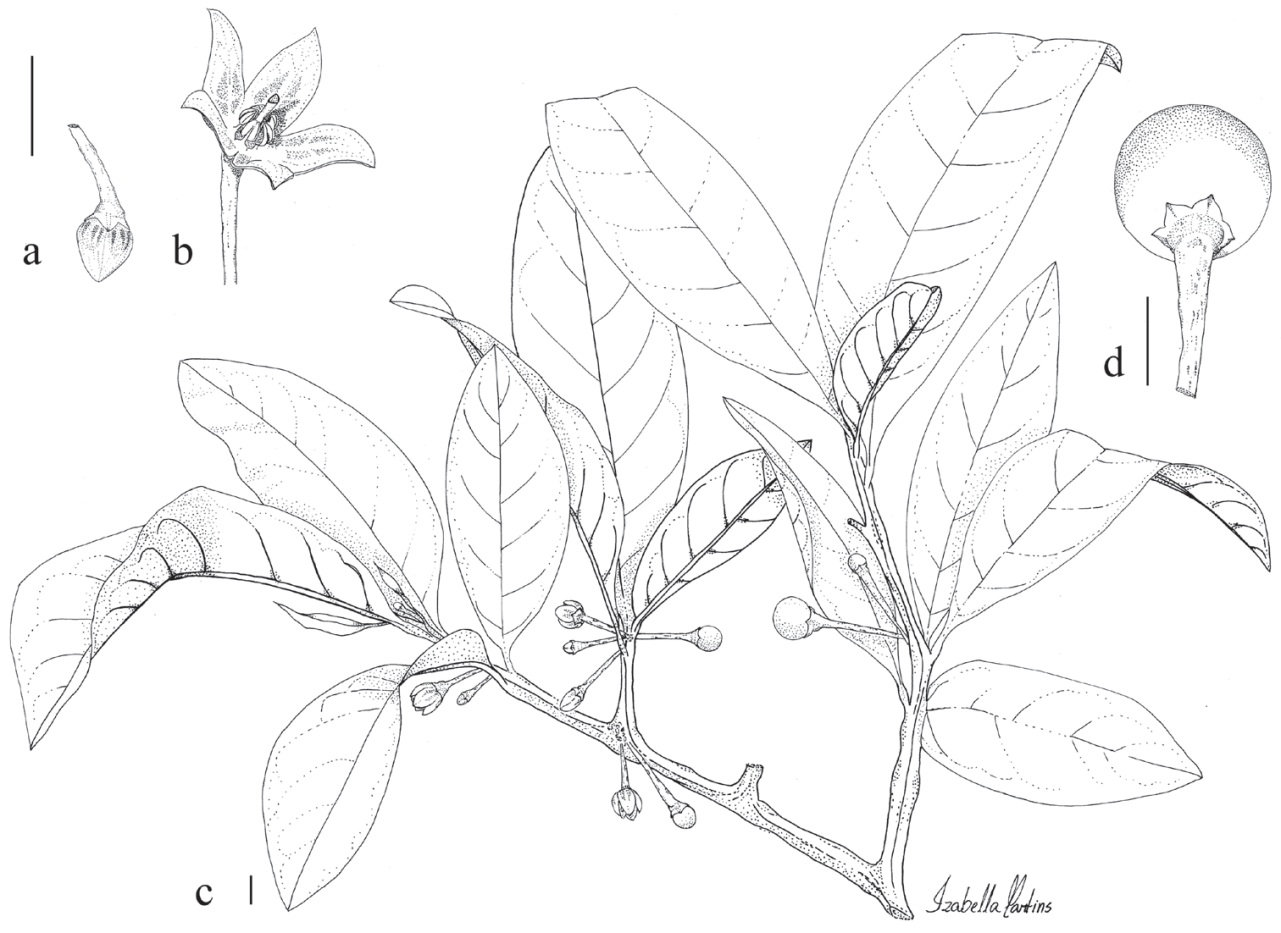
*Athenaea altoserranae* I.M.C. Rodrigues & Stehmann **a** flower bud **b** flower **c** branches with buds, flowers and fruits **d** fruit. Illustrated from *I.M.C. Rodrigues et al. 625* (BHCB). Illustration by I.M.C. Rodrigues. Scale bars 1 cm.

#### Type.

**Brazil. São Paulo**: Santo André, Distrito de Paranapiacaba, mais ou menos no km 3 da rodovia Estação de Campo Grande – Paranapiacaba, 18 Oct 1967 (fl, fr), *J. Mattos & N. Mattos 15078* (holotype: SP [acc. # 106084]).

**Figure 2.**
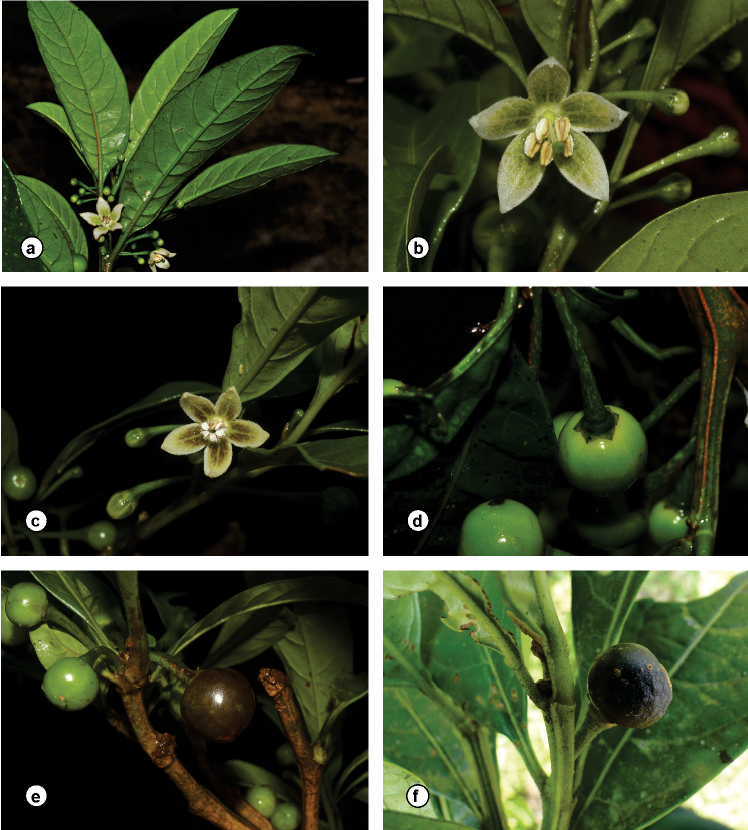
*Athenaea altoserranae* I.M.C. Rodrigues & Stehmann in the field **a***I.M.C. Rodrigues et al. 80***b, c, e***Stehmann et al. 4800* (BHCB) **d***I.M.C. Rodrigues et al. 83***f***I.M.C. Rodrigues et al. 625*. Photographs **a–e** by J.R. Stehmann **f** by S.N. Moreira.

#### Description.

Small trees, 3–8 m tall; branching dichotomous, especially distally. Stems quadrangular in cross-section, longitudinally striate, glabrous, brownish. Sympodial units difoliate, the leaves usually geminate, occasionally solitary. Leaves simple, alternate, spreading, arranged in clusters (short internodes) near branch apices, lanceolate, narrowly lanceolate, sometimes elliptic-lanceolate; blades (3.5–) 8.9–28.5 (50) cm long, (1)1.8–8.3 (15) cm wide, 2–6 times longer than wide, coriaceous, slightly discolorous; abaxial and adaxial surfaces glabrous, shiny; venation brochidodromous and prominent abaxially, with 6–12 pairs of principal veins; base attenuate, somewhat decurrent and asymmetric, occasionally somewhat cordate; margins entire and slightly revolute; apex acute or acuminate, sometimes notched (retuse); petioles (0.65–) 1–4(5.3) cm long, glabrous. Inflorescences axillary, sessile fascicles, with 4–8 flowers; pedicels 1.2–2.5 cm long, spreading or deflexed, glabrous and lenticellate, distally annular (constricted below a swollen pedicel apex). Flower buds globose, greenish-cream. Flowers 5-merous, heteromorphic with short- and long-styled forms. Calyx 1.8–3.5 mm long, campanulate-rotate, green, glabrous, the tube 0.4–1 mm long, the lobes 1–2.5 mm long, 2–3 mm wide, triangular, the apex acute or obtuse, glabrous on both surfaces. Corolla 7–13 mm long, stellate, white abaxially, cream with green to brownish spots adaxially, the tube 3–4 mm long, the lobes 3.5–9 mm long, 3–5.6 mm wide, triangular-lanceolate, glabrous abaxially, with glandular trichomes (stalk 2-celled, the head unicellular) at the base adaxially, the margins densely papillate. Stamens equal, glabrous; filaments 1.8–3 mm long, the stapet (basal extension of the filaments) ca. 1.5 mm long; anthers 1.8–3 mm long, 1–1.5 mm wide, oblong, yellowish-green, dehiscent by longitudinal slits. Ovary ca. 2.5 mm long, subglobose, greenish-cream, glabrous, surrounded by a yellowish green nectary; styles heteromorphic, long styles 4.2–5 mm long, short styles 2.3–2.8 mm long, greenish-white; stigma dilated, discoidal, greenish-white. Fruit a subglobose berry, 8–16 mm long, 8–19 mm in diameter, green when immature, dark brown to almost purplish-black at maturity, the surface glabrous; fruiting calyx not accrescent, up to 1.8 times longer in fruit than in flower, the apex of lobes up to 1/4 of fruit length; fruiting pedicels 2.1–4.1 cm long, enlarged at the apex, glabrous, pendent. Seeds up to 22 per berry, 2.4–2.8 mm long, 2.2–2.5 mm wide, semicircular to ovoid-reniform, yellow to brown, the testa reticulate, foveolate, the embryo curved. Chromosome number: not known.

**Figure 3.**
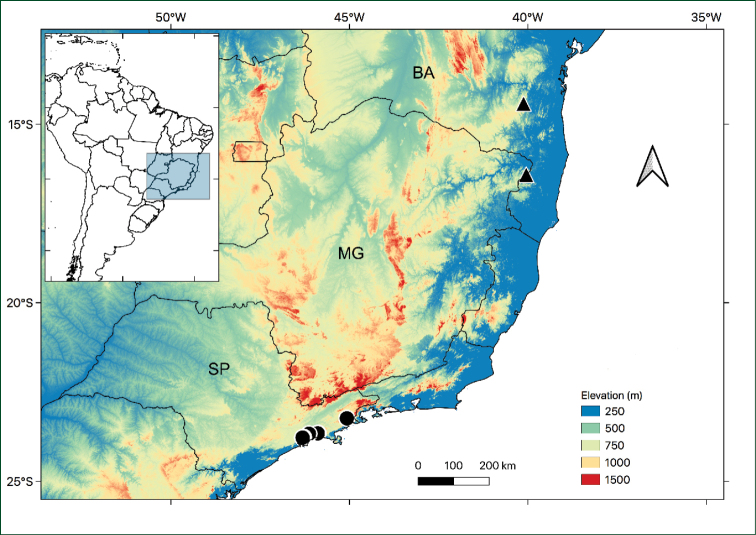
Distribution map for *Athenaea altoserranae* I.M.C. Rodrigues & Stehmann (circles) and *Athenaea hunzikeriana* I.M.C. Rodrigues & Stehmann (triangles). Bahia = BA; Minas Gerais = MG; São Paulo = SP.

#### Etymology.

The specific epithet is a reference to “Alto da Serra”, located in the District of Paranapiacaba, Municipality of Santo André, São Paulo State, where the species, including the type gathering, has been collected since 1917. This area was the first biological station in South America, established in 1909 by Hermann Friedrich Albrecht von Ihering. In 1938, under the administration of the Instituto de Botânica de São Paulo, the name was changed to Reserva Biológica do Alto da Serra de Paranapiacaba (Marcolin 2009).

#### Phenology.

*Athenaea altoserranae* flowers and fruits from August to March.

#### Distribution and habitat.

*Athenaea altoserranae* is endemic to Brazil and restricted to the edges of the plateau of the Serra do Mar mountain range in São Paulo State. It grows in the wet Atlantic rain forest (Mata Atlântica), often in clearings and along trails or other open places from 714 to 1,100 m elevation.

#### Preliminary conservation assessment

(IUCN 2019). Endangered (EN). EOO 1,240.9 km^2^ (EN); AOO 48 km^2^ (EN). This species occurs in the plateau of Serra do Mar and near the coast in the Municipality of Cananéia, São Paulo State, an area originally totally covered by Atlantic rain forest. The restricted range, coupled with the fragmented habitat, suggests a preliminary assessment of Endangered B1a and B2a, b (ii and iii).

#### Discussion.

Specimens of *Athenaea altoserranae* have been variously identified in herbaria as *Aureliana fasciculata* (Vell.) Sendtn., Aureliana fasciculata var. longifolia (Sendtn.) Hunz. and Barboza or *Aureliana glomuliflora* Sendtn. – all currently recognised as synonyms of the widespread glabrous species *Athenaea fasciculata* (Vell.) I.M.C. Rodrigues & Stehmann. *Athenaea fasciculata* is the most widespread species of the genus and is distributed along the Atlantic rain forest from southern to north-eastern Brazil, but also occurs south to Paraguay and Argentina and west to eastern Peru and Bolivia (see Rodrigues et al. 2019). *Athenaea altoserranae* is sympatric with *A. fasciculata*, *A. cuspidata*, *A. pogogena*, *A. picta*, *A. sellowiana* and *A. wettsteiniana*.

Hunziker and Barboza (1990) recognised three infraspecific taxa within their circumscription of *Aureliana fasciculata* (A. fasciculata var. fasciculata, A. fasciculata var. longifolia Sendtn. and A. fasciculata var. tomentella Hunz. & Barboza). They included material of Athenaea altoserranae in their circumscription of var. longifolia along with other specimens collected in Bahia and the border of São Paulo with Paraná (SP/PR); the only original material we have seen for var. longifolia was destroyed in the bombing of the Berlin herbarium and no duplicates have been found, but a photograph (F neg. 2880, of an un-numbered F. Sellow collection from “Brasilia australiore” annotated “*Bassovia glomuliflora* Dun. β *longifolium*”) corresponds to *A. fasciculata* s.l. and not to *A. altoserranae* (Rodrigues et al. 2019). Although all of the specimens included in var. longifolia by Hunziker and Barboza (1990) share completely glabrous leaves, the plants from Bahia and from the São Paulo/Paraná border have small flowers, long-obovate leaves with a cuspidate apex and longer petioles, thus distinguishing them from the plants here recognised as *A. altoserranae*. We currently include these plants from Bahia and from the São Paulo/Paraná border in our circumscription of *A. fasciculata* s.l.

*Athenaea altoserranae* can be distinguished from *A. fasciculata* s.l. by its erect and long-lanceolate leaves clustered at the tips of shoots versus broader leaves evenly spaced along the stem, warty versus smooth fruiting pedicels and dark brown versus green mature fruits. Phylogenetic studies of the group, using molecular data (plastid and nuclear markers), provide additional support for the distinction of these populations. Zamberlan et al. (2015) found that *Athenaea altoserranae* (as *Aureliana* sp. nov.) does not belong to the same clade as *Aureliana fasciculata*, but is, instead, a member of a clade with *A. sellowiana* (Sendtn.) I.M.C. Rodrigues & Stehmann and *A. velutina* (Sendtn.) D’Arcy that is sister to *Athenaea pogogena* (Moric.) Sendtn. + *Athenaea cuspidata* Witasek +*Athenaea picta* (Mart.) Sendtn.

*Athenaea sellowiana* is also morphologically similar to *A. altoserranae* in its glabrous stems and leaves and is broadly sympatric with it in São Paulo State. The two species can be distinguished by flower texture (calyx and corolla fleshy in *A. sellowiana* and foliaceous in *A. altoserranae*) and the more strictly lanceolate leaves of *A. altoserranae* (versus narrowly oblanceolate, elliptic or oblong leaves in *A. sellowiana*).

#### Additional specimens examined.

**Brazil. São Paulo**: Cunha, Parque Estadual da Serra do Mar, 12 Dec 1989 (fl), *O.T. Aguiar 338* (SPSF); Cunha, Parque Estadual da Serra do Mar, Estrada de acesso ao Núcleo, margens do Rio Paraibuna, 28 Jan 2004 (fl, fr), *F.A.R.D.P. Arzolla 435* (ESA); Biritiba Mirim, Estação Biológica de Boracéia, 29 Sep 1983 (fl), *A. Custódio Filho 1570* (BHCB); Biritiba Mirim, Estação Biológica de Boracéia, 29 Sep 1983 (fl), *A. Custódio Filho 1592* (SP); Biritiba Mirim, Estação Biológica de Boracéia, 21 Oct 1983 (fl), *A. Custódio Filho 1723* (SP); Biritiba Mirim, Estação Biológica de Boracéia, 26 Oct 1983 (fl, fr), *A. Custódio Filho 1735* (SP); Alto da Serra, 20 Nov 1984 (fl), *T.P. Guerra & M. Kirizawa 90* (BHCB); Alto da Serra, Caixa d’água, 6 Oct 1922 (fl), *F.C. Hoehne s.n.* (SP); Natividade da Serra, Parque Estadual da Serra do Mar, Núcleo Santa Virgínia, 14 Nov 2005 (fl, fr), *N.M. Ivanauskas et al. 5196* (MBM); Bertioga, Rodovia BR 101, Km 216, 21 Aug 1995 (fl, fr), *M. Kirizawa et al. 3177* (BHCB); Cunha, Parque Estadual da Serra do Mar, trilha para a casa do Silvestre, 20 Mar 1996 (fr), *M. Kirizawa et al. 3271* (SPF); Alto da Serra, Estação Biológica, 17 Mar 1944 (fr), *M. Kuhlmann s.n.* (SP); Salesópolis, Boracéia, 22 Nov 1957 (fl), *M. Kuhlmann 4308* (SP); Estação Biológica do Alto da Serra de Paranapiacaba, 27 Nov 1980 (fl), *E.A. Lopes et al. 92* (SP); Cunha, Parque Estadual da Serra do Mar, trilha do Rio Bonito, 16 Nov 2006 (fl, fr), *E.J. Lucas et al. 369* (BHCB); Santo André, A. da Serra, Est. Biológica, 19 Oct 1918 (fl), *A. Pedroso 13* (SP); Salesópolis, Estação Biológica de Boracéia, 20 Oct 2001 (fl, fr), *J.R. Pirani et al. 4906* (SP); Distrito de Paranapiacaba, Estrada para a parte alta do distrito, em frente à guarita da Estação Biológica do Instituto de Botânica, 12 Oct 2009 (fl, fr), *I.M.C. Rodrigues et al. 71* (BHCB); Caminho da Bela Vista, Parque Ecológico Municipal das Nascentes de Paranapiacaba, 13 Oct 2009 (fl, fr), *I.M.C. Rodrigues et al. 75* (BHCB); Caminho da Bela Vista, 13 Oct 2009 (fl, fr), *I.M.C. Rodrigues et al. 76* (BHCB); Caminho da Bela Vista, Parque Ecológico Municipal das Nascentes de Paranapiacaba, 13 Oct 2009 (fl), *I.M.C. Rodrigues et al. 77* (BHCB); Caminho da Bela Vista, Parque Ecológico Municipal das Nascentes de Paranapiacaba, 13 Oct 2009 (fl, fr), *I.M.C. Rodrigues et al. 80* (BHCB); Caminho da Bela Vista, Parque Ecológico Municipal das Nascentes de Paranapiacaba, 13 Oct 2009 (fl, fr), *I.M.C. Rodrigues et al. 81* (BHCB); Caminho da Bela Vista, Parque Ecológico Municipal das Nascentes de Paranapiacaba; 13 Oct 2009 (fl, fr), *I.M.C. Rodrigues et al. 82* (BHCB); Caminho da Bela Vista, Parque Ecológico Municipal das Nascentes de Paranapiacaba; 13 Oct 2009 (fl, fr), *I.M.C. Rodrigues et al. 83* (BHCB); Santo André, Distrito de Paranapiacaba, em frente à guarita da Reserva Biológica do Instituto de Botânica de São Paulo, 23 Jan 2014 (fl, fr), *I.M.C. Rodrigues et al. 625* (BHCB); Cananéia, Ilha do Cardoso, Reserva Biológica, Margem do Rio Perequê, 14 Sep 1983 (fl), *S. Romaniuc Neto 97* (SP); Paranapiacaba, 3 Oct 1998 (fl), *L.C.Q.M.P. Sampaio & D. Vedovello 119* (BHCB); Santo André, Alto da Serra, Dec 1917 (fl), *E. Schwebel 132* (SP); Mogi das Cruzes, Sítio do Mauro Peixoto, 24 Oct 2007 (fl, fr), *J.R. Stehmann et al. 4800* (BHCB); Biritiba Mirim, Estação Biológica de Boracéia, 24 Oct 2007 (fl), *J.R. Stehmann et al. 4818* (BHCB); Biritiba Mirim, Estação Biológica de Boracéia, 24 Oct 2007 (fl, fr), *J.R. Stehmann et al. 4819* (BHCB); Biritiba Mirim, Estação Biológica de Boracéia, 24 Oct 2007 (fl, fr), *J.R. Stehmann et al. 4820* (BHCB); Biritiba Mirim, Estação Biológica de Boracéia, 24 Oct 2007 (fl, fr), *J.R. Stehmann et al. 4823* (BHCB); Biritiba Mirim, Estação Biológica de Boracéia, 24 Oct 2007 (fl), *J.R. Stehmann et al. 4824* (BHCB); Biritiba Mirim, Estação Biológica de Boracéia, 24 Oct 2007 (fl), *J.R. Stehmann et al. 4825* (BHCB); Biritiba Mirim, Estação Biológica de Boracéia, Estrada para Salesópolis, 24 Oct 2007 (fl, fr), *J.R. Stehmann et al. 4839* (BHCB); Cunha, Parque Estadual da Serra do Mar, 24 Oct 2007 (fl), *J.R. Stehmann et al. 4846* (BHCB); Cunha, Parque Estadual da Serra do Mar, 24 Oct 2007 (fl), *J.R. Stehmann et al. 4848* (BHCB); Santo André, Estrada Paranapiacaba – Taquarussú, 14 Oct 2009 (fl, fr), *V.A. Thode et al. 251* (BHCB); Santo André, Estrada Paranapiacaba – Taquarussú, 14 Oct 2009 (fl, fr), *V.A. Thode et al. 253* (BHCB); Santo André, Estrada Paranapiacaba – Taquarussú; 14 Oct 2009 (fl, fr), *V.A. Thode et al. 254* (BHCB).

### Athenaea hunzikeriana

Taxon classificationPlantaeSolanalesSolanaceae

I.M.C. Rodrigues & Stehmann sp. nov.

C131E62F-3EE5-5B11-ACB8-9E106C35B21A

urn:lsid:ipni.org:names:77217159-1

Figs 3 , 4 , 5

#### Diagnosis.

Similar to *A. pogogena*, but differing in the campanulate rather than inflated calyx and a subglobose (versus ovoid) fruit that is purplish-red when mature and pubescent with eglandular trichomes (versus green to yellow fruits with glandular trichomes).

#### Type.

**Brazil Minas Gerais**: Santa Maria do Salto, Fazenda Duas Barras, 16°24'45"S, 40°02'51"W, 817 m elev., 1 Nov 2013 (fl,fr), *J.R. Stehmann, L.L. Giacomin, S. Knapp & L. Bohs 6328* (holotype: BHCB [acc. # 182684; duplicates to be distributed to BM, RB).

**Figure 4.**
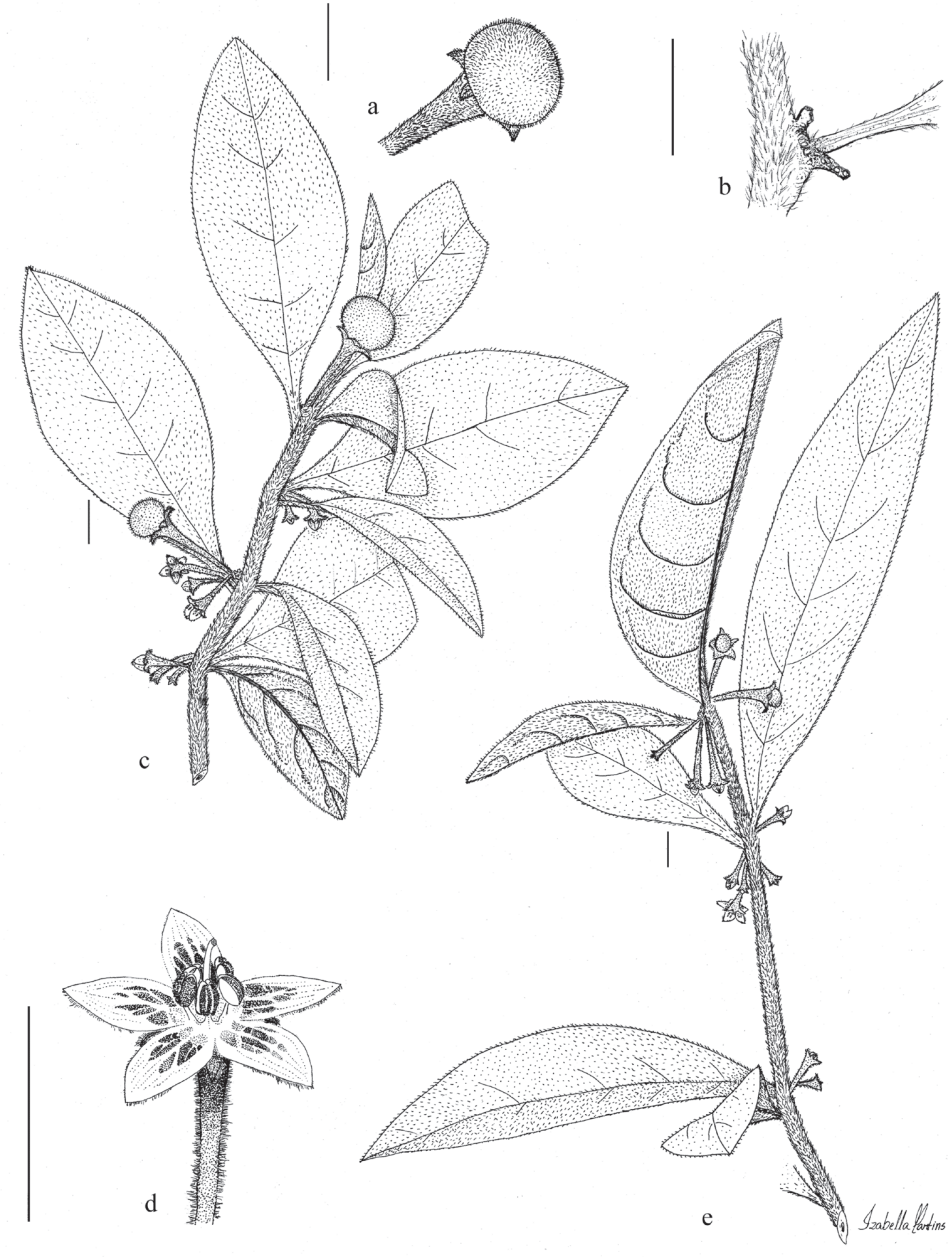
*Athenaea hunzikeriana* I.M.C. Rodrigues & Stehmann **a** fruit **b** stem showing reduced peduncle **c** branch with buds, flowers and fruits; illustrated from *J.A. Lombardi et al. 5447* (BHCB) **d** flower **e** branch with buds and flowers; illustrated from *J.R. Stehmann et al. 6328* (BHCB). Illustration by I.M.C. Rodrigues. Scale bars 1 cm.

#### Description.

Shrubs to small trees, up to 3 m tall; branching virgate and distally dichotomous. Stems rounded in cross-section, dark brown, tomentose with yellowish-brown, simple uniseriate trichomes, up to 2 mm long. Sympodial units difoliate, the leaves geminate, members of a pair markedly unequal. Leaves simple, alternate, elliptic to elliptic lanceolate, subcoriaceous, discolorous; blades of major leaves elliptic lanceolate, 8.7–16.4 cm long, 2.8–5 cm, 2.3–4.5 times longer than wide, the minor leaves elliptic to obelliptic 2.7–6.8 cm long, 1.3–3.2 cm wide, ca. 2.5 times longer than wide; abaxial and adaxial surfaces densely pubescent with 8–15-celled eglandular trichomes 0.5–2 mm long and scarce 1-celled glandular trichomes with usually 3-celled multicellular head; venation camptodromous and prominent abaxially, with 3–6 pairs of principal veins; base decurrent, the leaves appearing sessile or sub-sessile; margins entire and slightly revolute; apex acute to somewhat acuminate; petioles 0–0.8 cm long, densely pubescent with trichomes like those of the leaves. Inflorescences axillary, with 10–15 flowers along a distinct axis; rhachis 1.5–3.4 mm long; pedicels 0.6–2 cm long, erect to spreading, densely pubescent with simple uniseriate trichomes. Flower buds ovoid, white, densely glandular-pubescent. Flowers 5-merous, heteromorphic with long- and short-styled forms. Calyx 3.5–4.5 mm long, green, campanulate, densely pubescent, the tube ca. 2 mm long, the lobes 1.6–2.7 mm long, 0.8–1.4 mm wide, triangular, densely pubescent with 5–8-celled eglandular trichomes and longer 5–10-celled glandular trichomes with multicellular heads abaxially, sparsely pubescent with 4–8-celled glandular trichomes with multicellular heads adaxially, the lobe apices acute. Corolla 4.1–7.3 mm long, stellate, white with green and purple spots adaxially near the base, the tube 1.1–1.6 mm long, the lobes 3.4–5.6 mm long, 1.3–2.7 mm wide, lanceolate, covered with eglandular trichomes (3–4-celled) abaxially at the apex and 4–6-celled glandular trichomes with multicellular heads abaxially over the entire surface, adaxially with 3–5-celled glandular trichomes with unicellular heads. Stamens 5, equal, glabrous; filaments ca. 0.8 mm long, the stapet (basal extension of the filaments) ca. 0.6 mm long; anthers 1.4–1.9 mm long, 0.8–1 mm wide, oblong, yellowish-cream, slightly cordate at the base. Ovary ca. 0.6 mm long, subglobose, yellowish-cream, densely pubescent with glandular trichomes, surrounded by a yellowish-green nectary; styles heteromorphic, greenish-white, long styles 2.8–3 mm long, short styles ca. 2.2 mm long; stigma dilated, capitate, yellowish-white. Fruit a subglobose berry, ca. 12.6 mm long, 13.7 mm in diameter, green when immature, wine-red to purplish-red at maturity, the pericarp densely pubescent with simple, uniseriate 4–8-celled eglandular trichomes; fruiting calyx not accrescent, up to 1.8 times longer than in flower; fruiting pedicels 1.5–2 cm long, erect, enlarged at the apex, densely glandular-pubescent. Seeds ca. 6 per berry, 5.5–5.7 mm long, 3.8–4.0 mm wide, flattened-reniform, yellowish-brown, the testa minutely reticulate, foveolate, the embryo curved. Chromosome number: not known.

#### Etymology.

The specific epithet honours Ing. Armando Teodoro Hunziker (1919–2001), who dedicated his life to the study of the family Solanaceae in the Neotropics and whose mentorship formed a whole generation of Solanaceae workers.

#### Phenology.

*Athenaea hunzikeriana* flowers and fruits from August to November.

#### Distribution and habitat.

*Athenaea hunzikeriana* is endemic to Brazil and only known from the type locality, in north-eastern Minas Gerais State and in adjacent Bahia State. *Athenaea hunzikeriana* grows in the understorey of well-preserved remnants of wet Atlantic forests (Floresta Ombrófila Densa, Mata Atlântica; IBGE 2012), from 700 to 1000 m elevation.

#### Preliminary conservation assessment

**(IUCN 2019).** Data Deficient (DD). Just two populations of *A. hunzikeriana* are known, one growing in a private reserve (RPPN Fazenda Duas Barras), in the Municipality of Santa Maria do Salto, Minas Gerais and another in Fazenda Farofa in the Municipality of Boa Nova, Bahia. Although the locality in Bahia is well-protected, the extremely restricted range indicates the species is of some conservation concern.

#### Discussion.

*Athenaea hunzikeriana* is easily recognised by its purple, eglandular-pubescent mature fruits on erect or spreading pedicels, its large seeds and the distinctive inflorescences with a short but persistent axis. Other species of *Athenaea* have axillary fascicles, with no rhachis along which pedicel scars can be observed. This species is morphologically similar to and sympatric with *A. pogogena*, with which it shares almost all flower characteristics, but can be distinguished by its subglobose (versus conic or ovoid) berry and leaf pubescence of mostly eglandular trichomes with sparse minute glandular trichomes, rather than densely glandular pubescent leaves. *Athenaea hunzikeriana* is also vegetatively similar to *A. anonacea* Sendtn. and *A. martiana* Sendtn. in having pubescent leaves but is differentiated from both those species by its pubescent fruit and non-accrescent fruiting calyx. It is also sympatric with *A. fasciculata* but differs from that species in its conspicuous pubescence (*A. fasciculata* is glabrous, see discussion of *A. altoserranae* above).

**Figure 5.**
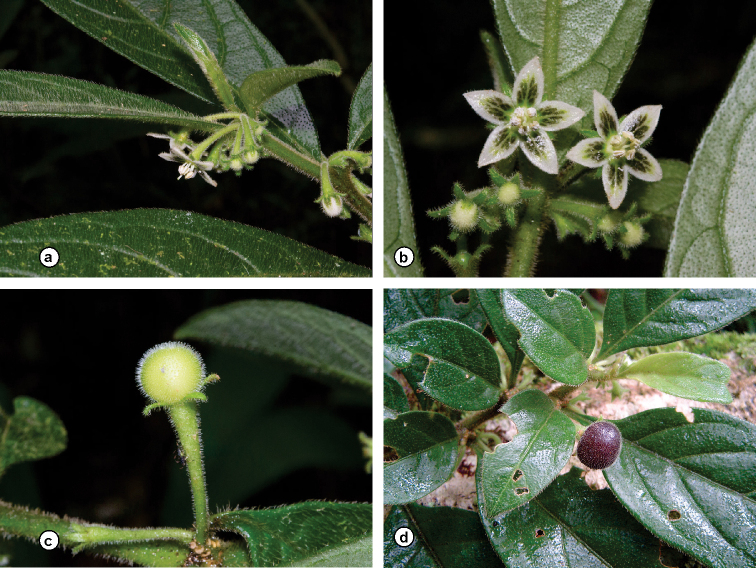
*Athenaea hunzikeriana* I.M.C. Rodrigues & Stehmann in the field **a–c***J.R. Stehmann et al. 6328***d***J.A. Lombardi et al. 5447* – All photographs by J.R. Stehmann.

#### Additional specimens examined.

**Brazil. Bahia**: Boa Nova, Faz. Farofa (Dr. Mauro), estrada entre Boa Nova e Dário Meira, 24 Oct 2007 (fr), *F.M. Ferreira 1641* (CEPEC). **Minas Gerais**: Santa Maria do Salto, Fazenda Duas Barras, 23 Aug 2003 (fl, fr), *J.A. Lombardi, J.R. Stehmann, R.C. Mota & L.G. Temponi 5447* (BHCB).

##### Dichotomous key to the species of *Athenaea*

All of the species in this key occur in Brazil, their distribution can be found in Rodrigues et al. (2019); *A. fasciculata* is the only species that occurs outside of Brazil.

**Table:** 

1	Leaves glabrous or with eglandular trichomes only	**2**
–	Leaves with glandular and eglandular trichomes	**10**
2	Leaves geminate, anisophyllous, usually sessile, with both surfaces hispid; fruiting calyx accrescent, the tip extending beyond the fruit length	***Athenaea martiana* Sendtn.**
–	Leaves solitary or geminate, not anisophyllous, subssessile or petiolate, glabrous to variously pubescent on both surfaces; fruiting calyx not or sometimes only partially accrescent, with the tip reaching up to 1/3 of fruit length	**3**
3	Stems, leaves, pedicels and calyx glabrous; leaves in clusters at branch tips	**4**
–	Stems, leaves, pedicels and calyx variously pubescent; leaves evenly spaced along branches, not clustered at branch tips	**5**
4	Leaves solitary, obovate-lanceolate; calyx more than 4 mm long; corolla thick and fleshy, with purple spots; fruits yellowish-green when mature	***Athenaea sellowiana* (Sendtn.) I.M.C. Rodrigues & Stehmann**
–	Leaves geminate, rarely solitary, elliptic to lanceolate; calyx less than 3.5 mm long; corolla not fleshy, with brownish spots; fruits dark brown when mature	***A. altoserranae* I.M.C. Rodrigues & Stehmann**
5	Leaves variously pubescent on both surfaces; calyx lobes triangular and of equal size and shape; fruits globose or subglobose	**6**
–	Leaves pubescent on the adaxial surface, hirsute on the abaxial surface; calyx lobes of unequal size and variously subulate; fruits ovoid	***Athenaea wettsteiniana* (Witasek) I.M.C. Rodrigues & Stehmann**
6	Leaves adaxially glabrescent, abaxially sparsely pubescent to tomentose; inflorescences with up to 12 flowers; fruiting calyx lobes equally divided	**7**
–	Leaves with both surfaces velvety pubescent; inflorescences generally with (6-) 13 to 26 flowers; fruiting calyx lobes unequally divided	***Athenaea velutina* (Sendtn.) D’Arcy**
7	Stems, abaxial leaf surfaces, pedicels and calyx tomentose; leaves ovate-elliptic, drying black; calyx tube narrowly urceolate in floral buds; calyx (tube+lobes) more than 3.5 mm long at anthesis	***Athenaea tomentosa* (Sendtn.) I.M.C. Rodrigues & Stehmann**
–	Stems, abaxial leaf surfaces, pedicels and calyx glabrescent to sparsely pubescent; leaves elliptic to lanceolate, drying pale green; calyx tube campanulate in floral buds; calyx (tube+lobes) up to 2.5 (-3) mm long at anthesis	**8**
8	Trichomes appressed, with broad bases and curved apices; calyx lobes with acute apices	***Athenaea brasiliana* Hunz.**
–	Trichomes patent, straight or curly; calyx lobes with obtuse to acute apices or with short caudate projections	**9**
9	Leaves narrowly lanceolate (length/width ratio > 4.8); corolla pubescent abaxially; seeds ca. 10 per fruit	***Athenaea angustifolia* (Alm.-Lafetá) I.M.C. Rodrigues & Stehmann**
–	Leaves elliptic, ovate to lanceolate (length/width ratio < 4.5); corolla glabrescent or glabrous abaxially; seeds more than 31 per fruit	***Athenaea fasciculata* (Vell.) I.M.C. Rodrigues & Stehmann**
10	Leaves sessile or subssessile; leaf apices acute; leaf bases decurrent; fruiting calyx not accrescent	**11**
–	Leaves long-petiolate, the petioles up to 9 cm long; leaf apices cuspidate, leaf bases attenuate; fruiting calyx accrescent	**12**
11	Stems dichotomously branching throughout, sometimes trichotomously branching; calyx lobes strongly unequal, with one or two longer than the others; fruits ovoid, glabrous, yellowish-green when mature	***Athenaea anonacea* Sendtn.**
–	Stems dichotomously branching only distally; calyx lobes equal; fruits subglobose, pubescent, vinaceous purple when mature	***Athenaea hunzikeriana* I.M.C. Rodrigues & Stehmann**
12	Calyx lobes with bases auriculate in flower and fruit; pedicels sparsely pubescent	***Athenaea cuspidata* Witasek**
–	Calyx lobes with bases not auriculate or only slightly cordate in fruit; pedicels densely pubescent	**13**
13	Trichomes of unequal size, arranged in two levels over the entire plant surface, translucent white; fruits glabrous; seeds more than 60 per fruit	***Athenaea picta* (Mart.) Sendtn.**
–	Trichomes of equal size, arranged in a single level over the entire plant surface, red-ferruginous; fruits pubescent; seeds up to 50 per fruit	***Athenaea pogogena* (Moric.) Sendtn.**

## Supplementary Material

XML Treatment for Athenaea altoserranae

XML Treatment for Athenaea hunzikeriana

## Supplementary materials

Supplementary material 1 GenBank accession numbers for *Athenaea altoserranae* I.M.C. Rodrigues & Stehmann Data type Genbank accession numbers Explanation note Complete Genbank accession number data for *Athenaea altoserrranae*, published as *Aureliana* sp. nov. in Zamberlan et al. (2015). https://binary.pensoft.net/file/542755This dataset is made available under the Open Database License (http://opendatacommons.org/licenses/odbl/1.0/). The Open Database License (ODbL) is a license agreement intended to allow users to freely share, modify, and use this Dataset while maintaining this same freedom for others, provided that the original source and author(s) are credited.Izabella Martins da Costa Rodrigues, Sandra Knapp, João Renato Stehmann

Supplementary material 2 Complete specimen details for *Athenaea altoserranae* and *Athenaea hunzikeriana* Data type specimens data Explanation note Table with habitat, loc notes, coordinates, altitude and specimen description notes of all examined specimens (also available on the NHM Data Portal as a dataset, https://doi.org/10.5519/zc80n093). https://binary.pensoft.net/file/542756This dataset is made available under the Open Database License (http://opendatacommons.org/licenses/odbl/1.0/). The Open Database License (ODbL) is a license agreement intended to allow users to freely share, modify, and use this Dataset while maintaining this same freedom for others, provided that the original source and author(s) are credited.Izabella Martins da Costa Rodrigues, Sandra Knapp, João Renato Stehmann

## References

[B1] Bachman S Moat J Hill A de la Torre J Scott B (2011) Supporting Red List threat assessments with GeoCAT: Geospatial conservation assessment tool. ZooKeys 150: 117–126. 10.3897/zookeys.150.2109 PMC3234434 22207809

[B2] Deanna R Larter M Barboza GE Smith SD (2019) Repeated evolution of a morphological novelty: A phylogenetic analysis of the inflated fruiting calyx in the Physalideae tribe (Solanaceae). American Journal of Botany 106(2): 270–279. 10.1002/ajb2.1242 30779447

[B3] Hunziker AT Barboza GE (1990) Estudios sobre Solanaceae XXX. Revisión de *Aureliana*. Darwiniana 30: 95–113.

[B4] IBGE – Instituto Brasileiro de Geografia e Estatística (2012) Manual técnico da vegetação brasileira 2 ed. Instituto Brasileiro de Geografia e Estatística, Rio de Janeiro, 271 pp.

[B5] IUCN (2019) Guidelines for Using the IUCN Red List Categories and Criteria. Version 14. Prepared by the Standards and Petitions Committee. [accessed 20.01.2020]

[B6] Marcolin N (2009) No topo da Serra. Revista Pesquisa FAPESP 166: 8–9. [accessed 10.05.2017]

[B7] Olmstead RG Bohs L Migid HA Santiago-Valentin E Garcia VF Collier SM (2008) A molecular phylogeny of the Solanaceae. Taxon 57(4): 1159–1181. 10.1002/tax.574010

[B8] Rodrigues IMC (2013) Estudos taxonômicos nos gêneros *Athenaea* Sendtn. e *Aureliana* Sendtn. (Solanaceae). PhD Thesis, Universidade Federal de Minas Gerais, Belo Horizonte, Brazil.

[B9] Rodrigues IMC Knapp S Stehmann JR (2019) The nomenclatural re-establishment of *Athenaea* Sendtn. (Solanaceae) with a nomenclatural synopsis of the genus. Taxon 68(4): 839–846. 10.1002/tax.12089

[B10] Thiers B (2019+) [continuously updated]: Index herbariorum: a global directory of public herbaria and associated staff. New York Botanical Garden’s virtual herbarium. [accessed 20.03.2016]

[B11] Zamberlan PM Rodrigues IMC Mäder G Castro L Stehmann JR Bonatto SL Freitas LB (2015) Re-evaluation of the generic status of *Athenaea* and *Aureliana* (Withaninae, Solanaceae) based on molecular phylogeny and morphology of the calyx. Botanical Journal of the Linnean Society 177(3): 322–334. 10.1111/boj.12246

