# Supplementary material for: Two new species of Athenaea Sendtn. (Solanaceae) from the Atlantic forests of south-eastern Brazil
Source: PhytoKeys. 2021 May 18;178:1–15. doi: 10.3897/phytokeys.178.64609 (PMC8149380; doi:10.3897/phytokeys.178.64609)
Supplement: Supplementary material 1 — GenBank accession numbers for Athenaea altoserranae I.M.C. Rodrigues & Stehmann [file phytokeys-178-001-s001.pdf]

Supplementary Material 1

GenBank accession numbers for *Athenaea altoserranae* I.M.C.Rodrigues & Stehmann

**Region**	**GenBank number**	**GenBank identification (Zamberlan et al. 2015)**	**Voucher specimen**
ndhF	KC549611	*Aureliana* sp. nov.	BHCB115939 (*Stehmann et al. 4820*)
trnL intron	KC549630	*Aureliana* sp. nov.	BHCB115939 (*Stehmann et al. 4820*)
trnL-trnF	KC549649	*Aureliana* sp. nov.	BHCB115939 (*Stehmann et al. 4820*)
psaI-accD	KC509483	*Aureliana* sp. nov.	BHCB115939 (*Stehmann et al. 4820*)
trnC-ycf6	KC509462	*Athenaea* sp. nov.	BHCB115939 (*Stehmann et al. 4820*)
ITS	KC832798	*Aureliana* sp. nov.	BHCB115939 (*Stehmann et al. 4820*)
